# First Report of a Toxic *Nodularia spumigena* (Nostocales/ Cyanobacteria) Bloom in Sub-Tropical Australia. I. Phycological and Public Health Investigations

**DOI:** 10.3390/ijerph9072396

**Published:** 2012-07-05

**Authors:** Glenn B. McGregor, Ian Stewart, Barbara C. Sendall, Ross Sadler, Karen Reardon, Steven Carter, Dan Wruck, Wasa Wickramasinghe

**Affiliations:** 1 Environment and Resource Sciences, Queensland Department of Science, Information Technology, Innovation and the Arts, 41 Boggo Road, Dutton Park, QLD 4102, Australia; 2 Queensland Health Forensic and Scientific Services, 39 Kessels Road, Coopers Plains, QLD 4108, Australia; Email: ian.stewart@griffith.edu.au (I.S.); barbara_sendall@health.qld.gov.au (B.C.S.); karen_reardon@health.qld.gov.au (K.R.); steve_carter@health.qld.gov.au (S.C.); 3 School of Public Health, Griffith University, Parklands Drive, Southport, QLD 4217, Australia; Email: ross.sadler@griffith.edu.au; 4 National Research Centre for Environmental Toxicology (EnTox), The University of Queensland, 39 Kessels Road, Coopers Plains, QLD 4108, Australia; Email: w.wickramasinghe@uq.edu.au

**Keywords:** cyanobacteria bloom, sub-tropical, cyanotoxin, nodularin

## Abstract

Cyanobacterial blooms represent one of the most conspicuous and widespread waterborne microbial hazards to human and ecosystem health. Investigation of a cyanobacterial bloom in a shallow brackish water recreational cable ski lake in south-eastern Queensland, Australia revealed the dominance of the toxigenic species *Nodularia spumigena*. The bloom spanned three months, during which time cell concentrations exceeded human guideline thresholds for recreational risk, and concentrations of the hepatotoxic cyanotoxin nodularin exceeded 200 µg L^−1^. Cyanotoxin origin and identification was confirmed by amplification of the *ndaF-*specific PCR product and sequencing of the 16S rRNA gene. From the limited data available leading up to, and throughout the bloom, it was not possible to establish the set of causative factors responsible for its occurrence. However a combination of factors including salinity, hydraulic retention time and nutrient status associated with an extended period of drought are likely to have contributed. This was the first known occurrence of this species in bloom proportions from sub-tropical Australia and as such represents a hitherto uncharacterized risk to human and ecosystem health. It highlights the need for adaptive monitoring regimes to ensure a comprehensive understanding of the potentially toxic cyanobacteria likely to inhabit any given region. Such monitoring needs to recognize that cyanobacteria have a significant capacity for range expansion that has been facilitated by recent changes in global climate.

## 1. Introduction

*Nodularia spumigena* Mertens ex Born. et Flah. is a brackish water heterocytous cyanobacterium known to form blooms in estuarine lakes in Europe, the Mediterranean, Canada, USA, South Africa, New Zealand and Australia. In Australia it occurs in estuaries [1,2,3], arid and semi-arid brackish inland lakes, and occasionally in the freshwater lakes of the lower Murray River in South Australia [4]. Typically it is restricted to the temperate regions of the continent where it has been known since the nineteenth century [5]. It produces the cyclic pentapeptide hepatotoxin nodularin [6] which, like the microcystins, strongly inhibits protein phosphatases 1 and 2A [7,8,9], and is a tumor promoter and suspected carcinogen [10]. Although no human fatalities have been attributed to nodularin intoxication, it has been associated with a number of stock and domestic animal poisoning events [11,12,13,14]. 

Monitoring and control measures targeting toxigenic cyanobacteria such as *N. spumigena* have, over past decades, focused on mitigating eutrophication as a key strategy e.g., [15,16,17,18]. However, changes in global climate systems and concomitant effects on aquatic environments have increasingly become a key aspect of bloom mitigation research e.g., [19,20]. Key climate drivers implicated in cyanobacterial bloom frequency and intensity in both freshwater and marine environments include changes in regional temperature regimes which affect species growth rates and lake stratification patterns [21], and altered hydrological cycles, as reflected in altered patterns of precipitation and drought periodicity [22,23,24]. In tropical and sub-tropical Australia, regional climate variability is strongly influenced by the behavior of the El Niño-Southern Oscillation (ENSO) [25]. Periods of extended El Niño activity are associated with cooling of sub-surface temperatures in the western Pacific and a decrease in average annual rainfall. In contrast, La Niña conditions are characterized by warming of western Pacific sea-surface temperatures and an increase in rainfall over eastern Australia. 

Much of the recent work relating cyanobacterial bloom occurrence to large scale climate change has been driven by observations that alterations of regional climate, notably minimum temperatures, have led to species range expansions. There are a number of well documented examples of this in recent years. One of these best examples is the toxigenic species, *Cylindrospermopsis raciborskii*. Originally considered to occupy a pan-tropical distribution, since the early decades of the 20th century there have been increasing records of its occurrence at higher latitudes throughout Europe and North America [26,27,28,29]. These cases highlight the need for ongoing and adaptive monitoring regimes to ensure a comprehensive understanding of the potentially toxic cyanobacteria likely to inhabit any given habitat. Such monitoring needs to recognize that cyanobacteria have a significant capacity for range expansion that has been facilitated by recent changes in global climate.

During the spring of 2008, a dense cyanobacterial bloom was observed in a shallow brackish water recreational cable ski lake adjacent to the Logan River, south-east Queensland. A subsequent investigation revealed that the predominant organism was the potentially toxic cyanobacterium *N. spumigena*. This was the first known occurrence of this species in bloom proportions from sub-tropical Australia and as such represented a hitherto uncharacterized risk to human and ecosystem health. An integrated polyphasic approach based on morphological, molecular and toxicological characterization was applied to assess the health risk posed to lake users from the bloom.

## 2. Results and Discussion

### 2.1. Identification of N. spumigena

**Figure 1:**
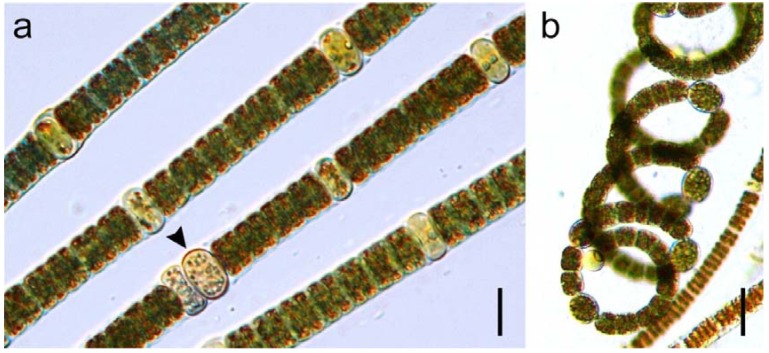
(**a**) *Nodularia spumigena*, arrow indicates akinetes (scale bar 10 µm); (**b**) *Anabaenopsis arnoldii* (scale bar 15 µm).

The bloom material observed showed morphological features characteristic of *N. spumigena* [30]. Trichomes were solitary, cylindrical, straight to slightly flexuous and generally >500 µm in length (Figure 1). Vegetative cells were discoid 7.8–9.5 µm × 2.5–3.9 µm, containing many aerotopes. Heterocytes were also compressed discoid, 9.8–11.3 µm × 4.6–6.7 µm, intercalary and regularly spaced. Akinetes were common, discoid-subspherical, 10.6–11.7 µm × 4.8–8.0 µm, mostly single or in pairs, occasionally 3–5 in series.

### 2.2. Phytoplankton

**Figure 2:**
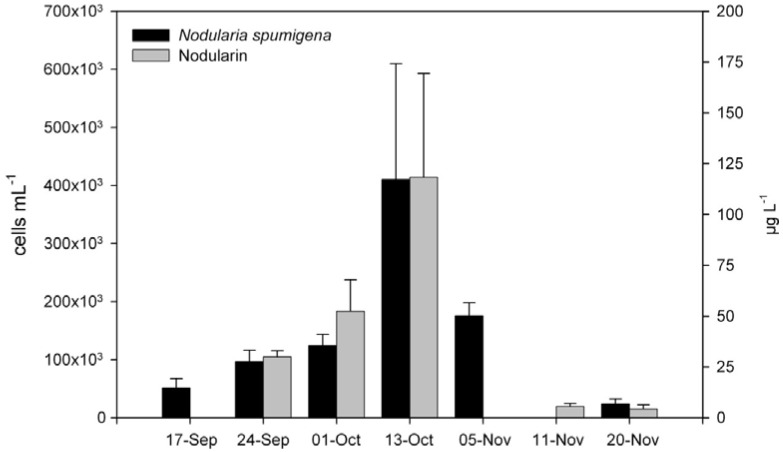
Temporal changes in *Nodularia spumigena* cell concentration and nodularin concentration in Carbrook Cable Ski Lake during the bloom period (mean + SE).

Prior to the bloom, no routine phytoplankton monitoring was undertaken in the ski park lake; therefore it is possible that *N. spumigena* was present before the bloom was visually detected. The bloom was originally observed as a thick bright blue-green surface scum concentrated by wind-driven advection along the lake shore. The cyanobacteria population gradually increased throughout September, peaking in mid-October at a total cell biovolume of 802 mm^3^ L^−1^, before rapidly falling below detection limits at the end of November (Figure 2). The phytoplankton during this time was dominated by *N. spumigena*, which on average, represented >90% of the total phytoplankton biovolume. *Anabaenopsis arnoldii* Aptekarj (Figure 1b) was subdominant throughout the bloom and co-occurred with a number of diatom species, notably *Chaetoceras* spp.

### 2.3. Toxin in Water

The concentration of nodularin was closely correlated with the concentration of *N. spumigena* cells (Figure 2), indicating that most of the toxin present was in the intracellular phase. The highest concentration of nodularin recorded was 220 µg L^−1^ at the peak of the bloom, corresponding to the highest *N. spumigena* cell concentrations between 164,500–605,200 cells mL^−1^.

### 2.4. Toxin in Isolated Strains of N. spumigena

Nodularin was detected by HPLC in the strains of *N. spumigena* isolated from the lake, FSS1-112/1 and FSS1-115/1, and in the strain used as a positive control in PCR assays, CS-590/01. The strain CS-586/05 has been documented as non-producer of nodularin (also known as NSBL-05 in [31,32]) while CS-590/01 is a known nodularin-producer (also known as NSLA-01 in [31,32]). However, CS-586/05 was found to produce nodularin at the limit of detection for the assay of 0.1 mg L^−1^ (results not shown). The *ndaF* gene was also amplified from this strain, confirming that CS-586/05 has one of the genes necessary for nodularin production.

### 2.5. Molecular Confirmation of Toxin Origin and Identification of N. spumigena

The presence of a nodularin-producing population of *N. spumigena* in the bloom was confirmed by amplification of the *ndaF*-specific PCR product of approximately 189 bp. Isolation of *N. spumigena* strains FSS1-112/1 and FSS1-115/1 from two water samples from the lake, followed by detection of the *ndaF* gene and confirmation of the ability of the strains to produce nodularin, added further evidence that *N. spumigena* was the origin of the toxin detected in the lake. Amplicons of *ndaF* were present in *N. spumigena* CS-586/05 and CS-590/1 but were absent from *C. raciborskii* FSS1-127/1 and *M. aeruginosa* CS-558/01.

Sequencing of the *ndaF* PCR products from the bloom sample, FSS1-112/1 and FSS1-115/1 (GenBank Accession numbers to be provided) confirmed they were 99% homologous to *ndaF* sequences in GenBank (for example, AY382542, AY382540, AJ781160). Additionally, the sequences of the *ndaF* amplicons from the bloom sample and the two FSS strains were identical to each other (data not shown).

A partial 791 bp sequence of the 16S rRNA gene from *N. spumigena* FSS1-115/1 was successfully sequenced (GenBank Accession JQ966090). Comparison with sequences obtained from GenBank demonstrated a high level of similarity (98–100% sequence identity; data not shown) with other *N. spumigena* sequences, including an Australian isolate NSKR07 from Western Australia (GenBank Accession AF268013; [32]). This very high level of similarity also extended to sequences from strains of *N. baltica*, (for example, AJ133177) *N. harveyana* (for example, AJ781143) and *N. sphaerocarpa* (for example, AJ781147). Sequencing data from the 16S rRNA gene of *N. spumigena* strain FSS1-115/1 from the lake confirmed the correct identification of this strain by morphometric analysis and also supported the morphological confirmation of the cultured strain.

### 2.6. Water Quality

The lake varied between eutrophic and hypereutrophic throughout the bloom with total phosphorus concentrations between 34–830 µg L^−1^ and total nitrogen concentrations between 1,833–3,800 µg L^−1^ (Table 1).

**Table 1:** Mean physico-chemical characteristics of Carbrook Cable Ski Lake during the period September–October 2008.

	Groundwater bore	Lake water			
Parameter	17 September	17 September	24 September	01 October	14 October
Conductivity	28.5	nd *	25	nd *	nd *
(mS cm^−1^)					
Total nitrogen	nd *	3,800	2,500	1,833	nd *
(µg L^−1^ as N)					
Total dissolved nitrogen	nd *	3,200	2,600	1,833	nd *
(µg L^−1^ as N)					
Ammonia nitrogen	4,000	1,400	653	10	nd *
(µg L^−1^ as N)					
Nitrogen oxides	3	20	68	7	nd *
(µg L^−1^ as N)					
Total phosphorus	nd *	180	51	34	830
(µg L^−1^ as P)					
Total dissolved phosphorus (µg L^−1^ as P)	nd *	40	50	33	nd *
Filterable reactive phosphorus (µg L^−1^ as P)	68	14	10	3	2

* nd—data not available.

From the limited physico-chemical data available leading up to, and throughout the bloom, it is not possible to establish the set of causative factors responsible for bloom initiation and eventual collapse, or to quantify the history of previous occurrences. However, examination of historical aerial satellite imagery suggests that this incident is not an isolated one. *Nodularia spumigena* has a discrete salinity preference range. Salinity was shown to have a significant effect on the growth and toxin production of a *N. spumigena* strain from the Baltic Sea, with optimal salinity between 11–26.5 mS cm^−1^ [33]. However in Australia, *N. spumigena* has been reported at much broader salinity ranges. Blooms of *N. spumigena* occur frequently in Lakes Alexandrina and Albert in South Australia [4]. Although at the time of Francis [5] these lakes were brackish, due to extensive river regulation over the past century, these lakes are now much fresher with conductivity concentrations on average 0.532 mS cm^−1^ [34]. At the time of the Carbrook lake bloom, south-east Queensland was experiencing an extended period of drought (1995–2009), which was dominated by El Niño conditions, and characterized by below average rainfall. This extended dry period is likely to have increased the lake salinity, with evaporation exceeding precipitation, and the contribution of brackish groundwater increased in relation to local catchment runoff to maintain lake water levels. Under these drought conditions, the lake water residence time and nutrient concentrations increased over time. The abundance of large bloom-forming cyanobacteria, such as *N. spumigena*, has shown a positive relationship to hydraulic residence time [35,36] which is likely to be related to their slower reproductive rates compared to other smaller planktonic algae [37]. Although hydraulic retention time is a good predictor of bloom frequency, nutrient status is an important determinant of bloom intensity. A combination of factors including salinity concentration, hydraulic retention time and nutrient status which occurred in the Carbrook Lake at the time of the bloom was consistent with favorable growth conditions for *N. spumigena.*

Although *N. spumigena* has previously been reported sporadically as a minor component of the estuarine phytoplankton from sub-tropical regions of the continent, this is the first report of a toxic bloom of this magnitude. As such it represents a potential risk to both recreational water users and aquatic ecosystems within the adjacent receiving water system. During the bloom, cell biovolume levels exceeded the Australian Guidelines for Risks in Recreational Water [38] cyanobacteria red level action mode. The principal risk for recreational users of the lake was that of sudden immersion and ingestion of water contaminated by the cyanotoxin nodularin. The oral exposure route is the most hazardous; ingestion of a toxic dose results in fulminant hepatic failure. The kidney is also a target organ for nodularin. Some workers are investigating the potential for a related group of cyanotoxins (microcystins, a family of hepatotoxic cyclic heptapeptides) to be aerosolized, with implications for inhalational exposure, but that early work is inconclusive, and there are no anecdotal or case reports in either the human or veterinary literature that suggest toxic doses of cyanotoxins have been acquired solely by the inhalation route [39]. 

There are several reported incidents involving accidental ingestion of cyanotoxin-contaminated water following sudden immersion in recreational waters. Severe acute gastrointestinal and respiratory illnesses were described, and one fatality was investigated following horseplay-related forced immersion, though some diagnostic uncertainty surrounds that particular case [40]. A recent case report from Argentina describes a severe though self-limiting illness in a jet-skier following a two-hour exposure to microcystin-contaminated water in a hydroelectric reservoir. While a clinical diagnosis, as is often the case for such unusual and under-recognised exposures, the report is a graphic demonstration of the potential for cyanotoxins to accumulate in recreational waters at concentrations hazardous to human health. The affected teenager in this case required artificial ventilation in an intensive care unit; he also suffered acute renal and hepatic injury [41]. An earlier case report from the UK described hepatic dysfunction in a 39 year-old who was windsurfing in a microcystin-contaminated reservoir [42]. The specific activities at the cable ski park investigated in this study, in particular the use of ski jump ramps, would suggest a high likelihood of full immersion and accidental swallowing of water.

Nodularin is also a tumor promoter [10]. While this would normally be a concern only with respect to the potential for chronic, low-dose exposures through drinking water or consumption of contaminated seafood, the possibility of sub-acute but recurring exposures via inadvertent ingestion of nodularin-affected water in this particular recreational setting may deserve consideration. The lake business includes a ski club; members pay an annual fee which provides unlimited access. Regular visitors exposed to a long-standing *Nodularia* bloom may, in theory, experience sub-chronic exposure to nodularin. 

Nodularin can undergo trophic transfer; from a human health perspective the principal concerns relate to bioaccumulation in seafood and shellfish. This phenomenon is being actively researched in Baltic Sea food webs, with nodularin found in bivalves, benthic and pelagic fish and seabirds [43,44]. Commercial and recreational prawn and mussel harvesting in the Gippsland Lakes (Victoria, Australia) has been periodically restricted due to toxic *Nodularia* blooms [45]. Large mullet fish are present in the cable ski park that we studied. These fish are not harvested for consumption, and while the localized nature of this bloom should not result in widespread dissemination of nodularin into regional food webs, the possibility of isolated incidents of trophic transfer from these fish, or from smaller fish and invertebrates, into surrounding ecological niches should be considered. 

### 2.7. Public Health Response to the Toxic Nodularia Bloom

An *ad hoc* panel comprising public health and environment workers from Queensland Health, Logan City Council, Griffith University and the Queensland Department of Environment and Resource Management met on several occasions throughout the course of the bloom to consider *Nodularia* cell counts, nodularin concentrations and other site water quality data. The cable ski facility proprietor consented to suspend recreational access to the site on public health advice; operations resumed in late November 2008 when cell counts and toxin concentrations had declined to safe levels (Figure 2). During the voluntary closure, a period of approximately three months, the powered cable loop was inoperative and warning signs were erected at the site. Continuing operation of the facility following this toxic bloom was conditional on preparation and adoption of a specific management program, incorporating monitoring and reporting of cyanobacteria and other water quality parameters.

The economic burden of cyanobacteria blooms can be placed in the broader context of the economic impacts on freshwater systems of phenomena such as inorganic nitrogen pollution and eutrophication [6,17,46]. A comprehensive national or regional assessment of the cost of cyanobacteria blooms has not been conducted in any country to date. An analysis of freshwater eutrophication–cyanobacteria blooms being a particularly visible and tangible manifestation of this problem—in the U.S. put the cost at $2.2 billion annually [17]. In Australia, case studies from the 1991/92 summer season in New South Wales estimated losses to the tourism industry of between $1.2 million and $6.7 million from three discrete bloom events. An estimate based on a willingness to pay approach placed the annual economic impact of cyanobacteria blooms at $180 million to $240 million [47]. A comprehensive study of the economic cost would need to incorporate direct costs such as management of blooms (monitoring and treatment) by agencies responsible for recreational and drinking water storages [47]. Impacts on tourism accrue from public health interventions (warnings and closures) and loss of amenity caused by unsightly and malodorous blooms. Mass mortalities of fish and terrestrial animals will add further disincentives to visit. Property values may also be affected [17]. The agricultural sector can suffer significant stock losses due to toxic blooms [13,14,17]; cyanobacterial taste and odour compounds represent a considerable economic burden to the freshwater aquaculture industry [48,49].

While no formal economic assessment was conducted in relation to this *Nodularia* bloom, the proprietor of the cable ski business operating on the affected lake estimated a direct loss of some A$300K attributable to the bloom. These costs were accrued due to loss of income from the three-month closure of the operation on public health advice, and expenditure on interventions to disperse the bloom. This was a considerable financial burden on a business that is entirely dependent on public and member access to this particular waterbody, and the initiatives taken by the proprietor to protect his investment are instructive. During the course of the bloom, the proprietor sought advice on a range of interventional and preventive bloom remediation measures. He considered planting aquatic sedges such as *Phragmites* in order to sequester nitrogen and phosphorus; emergent macrophytes are an important feature of riparian buffers in natural and constructed wetlands [50]. In assessing the likelihood or otherwise of success through such an intervention, we suggested that planting emergent macrophytes would be unlikely to deliver a short-term solution for such a dense *Nodularia* bloom, and without a good understanding of nutrient sources and sinks in the lake, success over the longer term could not be guaranteed. Enriched sediments can contribute to high internal nutrient loads in freshwater waterbodies, which favors cyanobacterial chronicity [24]. Also the salinity of the lake would likely restrict the species range of emergent macrophytes that could be planted [51]. Application of copper sulfate as an algaecide was contemplated, but discouraged because of the likelihood of toxic effects on aquatic fauna in the lake and the nearby Logan River. An effective copper sulfate treatment would result in lysis of the bloom, which could conceivably exacerbate the immediate public health risks by liberating intracellular nodularin, thus increasing the concentration of free toxin in the water column [52]. The intervention eventually adopted by the cable ski lake operator at the time of the bloom was Phoslock^®^, a clay product that precipitates phosphorus and also forms a barrier on the sediment surface [24]. Three applications of Phoslock^®^ (15 tonnes, 10 tonnes and 2 tonnes) were made over a two month period. 

Beyond the short-term interventions taken to dissipate the bloom and facilitate the re-opening of his business, the cable ski operator is investigating the possibility of manipulating the physico-chemical parameters of his waterbody to prevent a recurrence of *Nodularia* blooms. He purchased a hobbyist microscope and a conductivity meter, and proceeded to research aspects of the ecology of *Nodularia*. He sunk a second bore, and by pumping brackish water from both bores into the lake, he demonstrated by regular conductivity measurements and record-keeping a small but steady rise in conductivity from 9‰ in December 2008 to 12‰ in September 2009. It is unclear at this stage, however, whether this approach will be able to alter long-term salinity levels in the lake to a degree sufficient to impede bloom-scale recurrences of *Nodularia*. 

## 3. Experimental Section

### 3.1. Study Area

Carbrook Cable Ski Lake (27°41′24′′S, 153°15′44′′E) is a small, shallow artificial lake adjacent to the Logan River, coastal south-east Queensland (Australia). It has a surface area of 4.9 ha and a mean depth of 3 m. The lake receives some surface runoff from the small local catchment; however the majority of the lake water is derived from regular supplementation by shallow brackish groundwater from an adjacent bore. This artificial recreational lake is one of two cable ski parks in south-east Queensland; the lessees of the business estimate that some 20% of park visitors could be described as inexperienced water skiers. A regular clientele attends the facility, with jump ramps and an obstacle course being major draw cards. The business attracts some 10,000 visits annually, with a typical summer Saturday hosting up to 60 attendees.

### 3.2. Phytoplankton Analysis

Sub-surface grab samples (depth < 0.5 m) were collected from three lake sites between September and November 2008 and fixed *in situ* with Lugol’s iodine to a final concentration of 1% prior to analysis. Identification and enumeration was performed microscopically using a calibrated glass Sedgwick-Rafter counting chamber [53]. A minimum of 30 fields and 100 units were counted to yield a final result of ±20% of the true cell concentration after [54]. Photomicrographs were taken using an Olympus DP12 digital microscope camera, and fifty morphological measurements of vegetative cells, heterocytes and akinetes were made from digital images of live material taken at ×400 magnification using UTHSCSA Image Tool V 3.0. Cell biovolume was calculated from cell dimensions applied to the formulae of [55].

### 3.3. Cyanotoxin Analysis

Nodularin was analysed in water by HPLC-PDA linked to an online concentrator. A 100 mL water sample was transferred to a disposable 100 mL plastic bottle and any cells present were lysed by sonication for one minute with a Branson Sonifier 450 (Branson Ultrasonics, Danbury, CT, USA). The sample was then passed through a 0.45 μM syringe filter and transferred to a 30 mL glass vial. The online concentrator HPLC system loads 25 mL sample onto a concentrator trap column consisting of a C18 Alltima guard cartridge 4.6 × 7.5 mm (W.R. Grace & Co., Columbia, MD, USA) which concentrates and washes the sample prior to the system eluting from the concentrator column into the analytical column of the HPLC system. Nodularin was identified with a photo-diode array detector using a UV max of 239 nm for quantitation. Samples were quantified against an in-house secondary standard of nodularin which was spiked into HPLC-grade reagent water and run on the online concentrator HPLC-PDA. The standard was certified using a UV spectrophotometer. The online concentrator HPLC-PDA system comprises a Prominence HPLC-PDA (Shimadzu Corp., Kyoto, Japan) fitted with a Luna C18 150 × 2 mm 5 µm column (Phenomenex, Torrance, CA, USA). The mobile phases were 5% acetonitrile and 95% acetonitrile; both mobile phases contained 8 mM ammonium acetate buffer. Analytical procedures were adapted from methods described by [56].

### 3.4. Isolation of N. spumigena from Bloom Samples

Two single trichome isolates of *N. spumigena* (FSS1-112/1 and FSS1-115/1) were isolated from water samples taken from the lake. FSS1-112/1 was isolated from the initial bloom sample sent to FSS for phytoplankton and toxin analysis while FSS1-115/1 was isolated from a lake sample received approximately four weeks after the initial bloom. In each case, a sub-sample of the environmental water was streaked onto the surface of MLA medium [57] solidified with agar (1% w/v) to separate out trichomes. Agar pieces bearing a single trichome of *N. spumigena* were identified by light microscopy excised and transferred to 24-well plates (ProScitech, Thuringowa, Australia) containing MLA medium. Where possible, single trichomes were transferred to the individual wells with an eyelash brush to minimize carry-over of agar. The well plates were incubated in a Binder growth chamber, with a 12:12 light:dark cycle between 10 and 30 mmol m^−2^ s^−1^, with a variable temperature setting synchronized with the light cycle; 24 °C light, 18 °C dark. Approximately 10–14 days later, the well plates were examined for growth and a subsample of each growing culture was transferred to individual plastic 25 cm^2^ vented tissue culture vessels. After a further 7–8 days of incubation, the cultures were transferred to and maintained in plastic 75 cm^2^ vented tissue culture vessels in a growth chamber as described.

### 3.5. Molecular Analysis

#### 3.5.1. DNA Extraction

DNA was extracted from 15 mL aliquots of water samples taken from the lake. Cyanobacterial cells were pelleted by centrifugation at 3,000 rpm for 20 min. The supernatant was removed; cell pellets were washed with 15 mL sterile Milli-Q water and centrifuged again. The supernatant was then removed as far as possible without disturbing the cell pellet. Cell pellets were stored at −20 °C until required. DNA was extracted from 100 µL cell pellets using a Qiagen DNeasy Blood and Tissue kit (Qiagen, Stockholm, Sweden).

DNA was extracted from the two strains of *N. spumigena* isolated at FSS and two strains of *N. spumigena* CS-586/05 and CS-590/01 obtained from the Australian National Algae Culture Centre. DNA was also isolated from non-nodularin producing strains *Cylindrospermopsis raciborskii* FSS1-127/1 and *Microcystis aeruginosa* CS-558/01. Aliquots of culture (1.5 mL) maintained in MLA were removed approximately 2–3 weeks after the strain had been sub-cultured. The culture was centrifuged at approximately 10,000 × g at room temperature in an Eppendorf MiniSpin Plus centrifuge (Lomb Scientific, Taren Point, Australia) for 3 min to pellet cyanobacterial cells. The supernatant was removed and DNA extracted using a Qiagen DNeasy Blood and Tissue kit.

#### 3.5.2. Amplification of the *ndaF* Gene Associated with Nodularin Biosynthesis

DNA extracted from the bloom sample and from pure cyanobacterial cultures was subjected to PCR amplification with primers specific to subunit F (*ndaF*) of the nodularin synthetase gene cluster [32]. The *ndaF* gene encodes two enzymes required for the production of nodularin, a polyketide synthase and a non-ribosomal peptide synthetase [58]. DNA from *C. raciborskii* FSS1-127/1 and *M. aeruginosa* CS-558 was included as negative controls for the *ndaF* gene as these strains produce cylindrospermopsin and microcystins, respectively. All PCRs were performed on a PerkinElmer GeneAmp^®^ PCR System 9700 thermocycler (Applied Biosystems, Foster City, CA, USA). Each 25 µL reaction comprised approximately 20 ng genomic DNA, 200 mM dNTPs (GE Healthcare Ltd., Chalfont St. Giles, UK), 2.5 mM MgCl_2_, 0.35 µM each primer (GeneWorks, Thebarton, Australia) and 1 U Ampli-Gold Taq DNA polymerase in 1 × PCR Buffer II (Applied Biosystems, Foster City, CA, USA). The cycling conditions comprised: initial denaturation at 95 °C for 10 min, followed by 35 cycles of 94 °C for 30 s, 50 °C for 30 s, 72 °C for 20 s. Cycling was followed by a final extension step at 72 °C for 10 min. 

#### 3.5.3. Amplification of the 16S rRNA Gene

A 791 bp region of the 16S rRNA gene was amplified with two different primer pairs: A2/409R-mod and 356F/PR1. The sequence of primers 356F and 409R is given in [59]; the sequence of 409R has been modified at the 3′ end, changing from a C to a T to enhance specificity (409R-mod). The sequence of A2 is given in [60], and the sequence for PR1 in [61]. PCR reactants were as described above. The cycling conditions comprised: initial denaturation at 95 °C for 10 min, followed by 28 cycles of 94 °C for 30 s, 55 °C for 30 s, 72 °C for 1 min. Cycling was followed by a final extension step at 72 °C for 10 min. For both the *ndaF* and 16S rRDNA amplicons, a 6–7 µL aliquot of the reaction was removed and visualized on a 1.5% agarose gel stained with ethidium bromide.

#### 3.5.4. Sequencing of *ndaF* and 16S rDNA Amplicons

Approximately 18 µL of PCR amplicons of the expected fragment size for the 16S rRNA and *ndaF* genes were purified using the Qiagen QIAquick PCR Purification Kit (Qiagen, Stockholm, Sweden) or treatment with phosphatase and exonuclease. Purified amplicons were sequenced with the Big Dye Terminator Cycle Sequencing Ready Reaction Kit version 3.1 (Applied Biosystems, Foster City, CA, USA) on an Applied Biosystems 3130 xl Genetic Analyser. Sequencing was performed at the Griffith University DNA Sequencing Facility (Brisbane, Australia) or by the Macrogen Standard Sequencing service (Macrogen, Seoul, South Korea). ChromasPro134 software (Technelysium Pty Ltd.) was used to generate consensus sequence data. Sequence alignments were made using the ClustalW algorithm in BioEdit v7.0.5.3 [62]. 

### 3.6. Chemical Analysis

Water samples were collected as sub-surface grabs at three sites throughout September–October 2008. Water samples for nutrient analyses were kept cool (<4 °C) in the dark prior to analysis. Analyses for filterable reactive phosphorus, nitrite, nitrate and ammonium were performed simultaneously using an automated LACHAT 8000QC flow injection system using methodology based on [63]. Samples for TN and TP were digested using a simultaneous persulfate digestion procedure based on that described by [63,64]. 

## 4. Conclusions

Cyanobacterial blooms remain a significant focus for health professionals due to their potential to impact on a range of environmental values. This was the first known occurrence of *N. spumigena* in bloom proportions from sub-tropical Australia, and highlights the need for phytoplankton monitoring regimes to be adaptive, recognizing the potential range expansion of toxigenic cyanobacteria facilitated by recent changes in global climate systems. Elucidating the causative factors for any given bloom and designing appropriate amelioration measures relies on collecting information on the physico-chemical drivers at appropriate spatial and temporal scales. Whilst this is common practice in high security water supply reservoirs, small-scale, private business owners such as the cable ski operator profiled in this investigation, rarely have the technical or financial resources to support such practices. Additionally, reservoir scale monitoring and management techniques do not easily translate to the recreational lake, farm dam or agricultural system scale. However the general phytoplankton monitoring principles and alert level frameworks outlined in the Australian Guidelines for Risks in Recreational Water [38] provide a robust basis for managing the risks of cyanobacterial blooms in recreational operations such as this. 

There are a number of potential cyanotoxin exposure pathways which need to be considered in recreational facilities such as the one profiled in this investigation. Whilst accidental ingestion, immersion through recreational exposure and aerosolized inhalation often receive the most attention, transfer and accumulation of cyanotoxins through regional food webs and subsequent ingestion through eating affected fish or shellfish is less commonly considered, particularly in the freshwater environment. 

Large mullet fish are present in the cable ski park that we studied. An initial investigation has revealed the accumulation of nodularin in the tissues of these fish. Results of that investigation are presented in the following paper as Part II of these studies into the first recorded bloom of toxic *Nodularia spumigena* in sub-tropical Australia. These results highlight the need for ongoing evaluation of exposure pathways to inform a comprehensive evaluation of the risks associated with *N. spumigena* blooms in this lake and similar waterbodies.

## References

[1] Blackburn S.I., McCausland M.A., Bolch C.J.S., Newman S.J., Jones G.J. (1996). Effect of salinity on growth and toxin production in cultures of the bloom-forming cyanobacterium *Nodularia spumigena* from Australian waters. Phycologia.

[2] Huber A.L. (1985). Factors affecting the germination of akinetes of *Nodularia spumigena* (Cyanobacteriaceae). Appl. Environ. Microbiol..

[3] John J., Kemp A. (2006). Cyanobacterial blooms in the wetlands of the Perth region, taxonomy and distribution: An overview. J. R. Soc. West Aust..

[4] Baker P.D., Humpage A.R. (1994). Toxicity associated with commonly occurring cyanobacteria in surface waters of the Murray-Darling basin, Australia. Aust. J. Mar. Freshw. Res..

[5] Francis G. (1878). Poisonous Australian lake. Nature.

[6] Camargo J.A., Alonso A. (2006). Ecological and toxicological effects of inorganic nitrogen pollution in aquatic ecosystems: A global assessment. Environ. Int..

[7] Eriksson J.E., Meriluoto J.A.O., Kujari H.P., Osterlund K., Fagerlund K., Hallbom L. (1988). Preliminary characterization of a toxin isolated from the cyanobacterium *Nodularia spumigena*. Toxicon.

[8] Yoshizawa S., Matsushima R., Watanabe M.F., Harada K., Ichihara A., Carmichael W.W., Fujiki H. (1990). Inhibition of protein phosphatases by microcystins and nodularin associated with hepatotoxicity. J. Cancer Res. Clin. Oncol..

[9] Honkanan R.E., Dukelow M., Zwiller J., Moore R.E., Khatra B.S., Boynton A.L. (1991). Cyanobacterial nodularin is a potent inhibitor of type 1 and type 2A protein phosphatases. Mol. Pharmacol..

[10] Ohta T., Sueoka E., Iida N., Komori A., Suganuma M., Nishiwaki R., Tatematsu M., Kim S., Carmichael W.W., Fujiki H. (1994). Nodularin, a potent inhibitor of protein phosphatases 1 and 2A, is a new environmental carcinogen in male F344 rat liver. Cancer Res..

[11] Nehring S. (1993). Mortality of dogs associated with a mass development of *Nodularia spumigena* (Cyanophyceae) in a brackish lake at the German North Sea coast. J. Plankton Res..

[12] Harding W.R., Rowe N., Wessels J.C., Beattie K.A., Codd G.A. (1995). Death of a dog attributed to the cyanobacterial (blue-green algal) hepatotoxin nodularin in South Africa. J. S Afr. Vet. Assoc..

[13] Van Halderen A., Harding W.R., Wessels J.C., Schneider D.J., Heine E.W., van der Merwe J., Fourie J.M. (1995). Cyanobacterial (blue-green algae) poisoning of livestock in the western Cape Province of South Africa. J. S. Afr. Vet. Assoc..

[14] Main D.C., Berry P.H., Peet R.L., Robertson J.P. (2008). Sheep mortalities associated with the blue green alga: *Nodularia spumigena*. Aust. Vet. J..

[15] Elmgren R., Larsson U. (2001). Nitrogen and the Baltic sea: Managing nitrogen in relation to phosphorus. Sci. World J..

[16] Conley D.J., Paerl H.W., Howarth R.W., Boesch D.F., Seitzinger S.P., Havens K.E., Lancelot C., Likens G.E. (2009). Controlling eutrophication: Nitrogen and phosphorus. Science.

[17] Dodds W.K., Bouska W.W., Eitzmann J.L., Pilger T.J., Pitts K.L., Riley A.J. (2009). Eutrophication of U.S. freshwaters: Analysis of potential economic damages. Environ. Sci. Technol..

[18] Petersen S.A. (1982). Lake restoration by sediment removal. J. Am. Water Resour. Assoc..

[19] Weidner C., Rücker J., Brüggemann R., Nixdorf B. (2007). Climate change affects timing and size of populations of an invasive cyanobacterium in temperate regions. Oecologia.

[20] Vilhena L.C., Hillmer I., Imberger J. (2010). The role of climate change in the occurrence of algal blooms: Lake Burragorang, Australia. Limnol. Oceanogr..

[21] Jöhnk K.D., Huisman J., Sharples J., Sommeijer B., Visser P.M., Stroom J.M. (2008). Summer heatwaves promote blooms of harmful cyanobacteria. Glob. Chang. Biol..

[22] Paerl H.W., Huisman J. (2008). Blooms like it hot. Science.

[23] Wagner C., Adrian R. (2009). Cyanobacteria dominance: Quantifying the effects of climate change. Limnol. Oceanogr..

[24] Paerl H.W., Hall N.S., Calandrino E.S. (2011). Controlling harmful cyanobacterial blooms in a world experiencing anthropogenic and climatic-induced change. Sci. Total Environ..

[25] Murphy R., Ribbe J. (2004). Variability of southeast Queensland rainfall and its predictors. Int. J. Climatol..

[26] Kling H.J. (2009). *Cylindrospermopsis raciborskii* (Nostocales, Cyanobacteria): A brief historic overview and recent discovery in the Assiniboine River (Canada). Fottea.

[27] Hamilton P.B., Ley L.M., Dean S., Pick F.R. (2005). The occurrence of the cyanobacterium *Cylindrospermopsis raciborskii* in Constance Lake: An exotic cyanoprokaryote new to Canada. Phycologia.

[28] Briand J.F., Leboulanger C., Humbert J.F., Bernard C., Dufour P. (2004). *Cylindrospermopsis raciborskii* (Cyanobacteria) invasion at mid-latitudes: Selection, wide physiological tolerance, or global warming?. J. Phycol..

[29] Padisák J. (1997). *Cylindrospermopsis raciborskii* (Woloszyńska) Seenayya et Subba Raju, an expanding, highly adaptive cyanobacterium: Worldwide distribution and review of its ecology. Arch. Hydrobiol..

[30] Komárek J., Hübel M., Hübel H., Smarda J. (1993). The *Nodularia* studies. 2. Taxonomy. Arch. Hydrobiol..

[31] Bolch C.J., Orr P.T., Jones G.J., Blackburn S.I. (1999). Genetic, morphological, and toxicological variation among globally distributed strains of *Nodularia* (Cyanobacteria). J. Phycol..

[32] Moffit C., Neilan B.A. (2004). Characterization of the nodularin synthetase gene cluster and proposed theory of the evolution of cyanobacterial hepatotoxins. Appl. Environ. Microbiol..

[33] Mazur-Marzec H., Zeglińska J., Pliński M. (2005). The effect of salinity on the growth, toxin production, and morphology of *Nodularia spumigena* isolated from the Gulf of Gdańsk, southern Baltic Sea. J. Appl. Phycol..

[34] Heresztyn T., Nicholson B.C. (1997). Nodularin concentrations in Lakes Alexandrina and Albert, South Australia, during a bloom of the cyanobacterium (blue-green alga) *Nodularia spumigena* and degradation of the toxin. Environ. Toxicol. Water Qual..

[35] Elliott A. (2010). The seasonal sensitivity of cyanobacteria and other phytoplankton to changes in flushing rate and water temperature. Glob. Chang. Biol..

[36] Carvalho L., Miller C.A., Scott E.M., Codd G.A., Davies P.S., Tyler A.N. (2011). Cyanobacterial blooms: Statistical models describing risk factors for national-scale lake assessment and lake management. Sci. Total Environ..

[37] Reynolds C.S. (2006). Ecology of Phytoplankton.

[38] National Health and Medical Research Council (NHMRC) (2008). Guidelines for Managing Risks in Recreational Water.

[39] Stewart I., Carmichael W.W., Sadler R., McGregor G.B., Reardon K., Eaglesham G.K., Wickramasinghe W.A., Seawright A.A., Shaw G.R. (2009). Occupational and environmental hazard assessments for the isolation, purification and toxicity testing of cyanobacterial toxins. Environ. Health.

[40] Stewart I., Webb P.M., Schluter P.J., Shaw G.R. (2006). Recreational and occupational field exposure to freshwater cyanobacteria—A review of anecdotal and case reports, epidemiological studies and the challenges for epidemiologic assessment. Environ. Health.

[41] Giannuzzi L., Sedan D., Echenique R., Andrinolo D. (2011). An acute case of intoxication with cyanobacteria and cyanotoxins in recreational water in Salto Grande Dam, Argentina. Mar. Drugs.

[42] Probert C.S., Robinson R.J., Jayanthi V., Mayberry J.F. (1995). Microcystin hepatitis. Arq. Gastroenterol..

[43] Ibelings B.W., Chorus I. (2007). Accumulation of cyanobacterial toxins in freshwater “seafood” and its consequences for public health: A review. Environ. Pollut..

[44] Sipiä V.O., Karlsson K.M., Meriluoto J.A., Kankaanpää H.T. (2004). Eiders (*Somateria mollissima*) obtain nodularin, a cyanobacterial hepatotoxin, in Baltic Sea food web. Environ. Toxicol. Chem..

[45] Van Buynder P.G., Oughtred T., Kirkby B., Phillips S., Eaglesham G., Thomas K., Burch M. (2001). Nodularin uptake by seafood during a cyanobacterial bloom. Environ. Toxicol..

[46] Stark C.H., Richards K.G. (2008). The continuing challenge of nitrogen loss to the environment: Environmental consequences and mitigation strategies. Dyn. Soil Dyn. Plant.

[47] Steffensen D.A. (2008). Economic cost of cyanobacterial blooms. Adv. Exp. Med. Biol..

[48] Smith J.L., Boyer G.L., Zimba P.V. (2008). A review of cyanobacterial odorous and bioactive metabolites: Impacts and management alternatives in aquaculture. Aquaculture.

[49] Tucker C.S. (2000). Off-flavor problems in aquaculture. Rev. Fish. Sci..

[50] Healy M., Cawley A.M. (2002). Nutrient processing capacity of a constructed wetland in western Ireland. J. Environ. Qual..

[51] Vymazal J. (2011). Plants used in constructed wetlands with horizontal subsurface flow: A review. Hydrobiologia.

[52] Jones G.J., Orr P.T. (1994). Release and degradation of microcystin following algicide treatment of a *Microcystis aeruginosa* bloom in a recreational lake, as determined by HPLC and protein phosphatase inhibition assay. Water Res..

[53] Hötzel G., Croome R. (1999). A Phytoplankton Methods Manual for Australian Freshwaters; Occasional Paper 22/29.

[54] Lund J.W.G., Kipling C., Le Cren E.D. (1958). The inverted microscope method of estimating algal numbers and the statistical basis for counting. Hydrobiologia.

[55] Hillebrand H., Dürselen C., Kirschtel D., Pollinger U., Zohary H. (1999). Biovolume calculation for pelagic and benthic microalgae. J. Phycol..

[56] Lawton L.A., Edwards C., Codd G.A. (1994). Extraction and high-performance liquid chromatographic method for the determination of microcystins in raw and treated waters. Analyst.

[57] Bolch C.J., Blackburn S. (1996). Isolation and purification of Australian isolates of the toxic cyanobacterium *Microcystis aeruginosa* Kütz. J. Appl. Phycol..

[58] Koskenniemi K., Lyra C., Rajaniemi-Wacklin P., Jokela J., Sivonen K. (2007). Quantitative real-time PCR detection of toxic *Nodularia* cyanobacteria in the Baltic sea. Appl. Environ. Microbiol..

[59] Neilan B.A., Jacobs D., Del Dot T., Blackall L.L., Hawkins P.R., Cox P.T., Goodman A.E. (1997). rRNA sequences and evolutionary relationships among toxic and non-toxic cyanobacteria of the genus *Microcystis*. Int. J. Syst. Bacteriol..

[60] Iteman I., Rippka R., Tandeau de Marsac N., Herdman M. (2002). rDNA analyses of planktonic heterocystous cyanobacteria, including members of the genera *Anabaenopsis* and *Cyanospira*. Microbiology.

[61] Shaw G.R., Sukenik A., Livne A., Chiswell R.K., Smith M.J., Seawright A.A., Norris R.L., Eaglesham G.K., Moore M.R. (1999). Blooms of the cylindrospermopsin containing cyanobacterium, *Aphanizomenon ovalisporum* (Forti), in newly constructed lakes, Queensland, Australia. Environ. Toxicol..

[62] Hall T.A. (1999). BioEdit: A user-friendly biological sequence alignment editor and analysis program for Windows 95/98/NT. Nucleic Acids Symp. Ser..

[63] Eaton A.D., Clesceri L.S., Rice E.W., Greenberg A.E., Franson M.A.H., American Public Health Association (APHA) (2005). Standard Methods for the Examination of Water & Wastewater.

[64] Hosomi M., Sudo R. (1986). Simultaneous determination of total nitrogen and total phosphorus in freshwater samples using persulfate digestion. Int. J. Environ. Stud..

